# Post COVID 19 economic recession shaping affective pathways of Gen Z’s environmental behavior across spheres of action

**DOI:** 10.1007/s43621-026-03982-4

**Published:** 2026-07-07

**Authors:** Shariq Waheed, Peter King, Husnain Waheed

**Affiliations:** 1 School of Sociology and Social Policy, University of Leeds, Leeds, LS2 9JT UK; 2 Sustainability Research Institute, School of Earth and Environment, University of Leeds, Leeds, LS2 9JT UK; 3 Department of Economics and Finance, Foundation University, Rawalpindi, Pakistan

**Keywords:** Resilience planning, Gen Z, Structural equation model, Environmental inequalities, Affective events theory, Spatio-economic transitions

## Abstract

The economic turbulence in the wake of the COVID-19 pandemic challenges public sector decision-makers in their efforts to sustain climate engagement. As economic and environmental challenges increasingly intersect, it becomes critical to explore youth’s environmental engagement during recession, particularly among Generation-Z, a cohort noted for its environmental-consciousness. Here, we used Affective Events Theory (AET) to develop and test an integrative-model exploring how the cognition of post-COVID-19 economic recession, in terms of its relevance and ability to cope, influenced Pro-Environmental Behaviors (PEBs) across multiple spheres. In doing so, the study extends AET beyond its established organizational and health applications to the economic-environmental nexus. A particular contribution of the model is the incorporation of financial-sphere PEBs, a dimension often sidelined for younger cohorts in behavioral research and policy discourses. We analyzed survey data from 320 Generation-Z respondents in six major Pakistani cities using Partial Least Squares Structural Equation Modelling and found that recession relevance amplifies both positive and negative affective reactions, while coping ability reduces positive but increases negative affect. These affective reactions mediate PEBs unevenly: strongest in public-sphere, while weakest in financial-sphere. Our results suggest that, to harness affective intensity during recessions for lasting sustainability gains, planners should channel youth emotions into structured forums such as climate-councils and community initiatives. Public engagement can be reinforced through civic-infrastructure, household and workplace practices through behavioral nudges, and financial-sphere engagement through microfinance, subsidies, and youth-friendly tariffs. Collectively, the findings offer insights for planners to ensure that economic adversity does not undermine Gen-Z’s environmental engagement.

**Supplementary Information:**

The online version contains supplementary material available at https://doi.org/10.1007/s43621-026-03982-4.

## Introduction

Economic turmoil has marked the post-COVID-19 era globally, with the pandemic precipitating the most severe recession since the Great Depression [1, 2]. While the global economy has shown signs of recovery from the initial shocks of the pandemic [3], the economic consequences have remained particularly severe across many developing countries, including Pakistan. The country experienced a contraction in GDP growth from 5.5% prior to the pandemic to −0.4% in 2020 [4], alongside escalating inflation that reached 19.9% in 2022 and contributed to a substantial increase in poverty levels [5, 6]. Pakistan was further confronted with a broader post-pandemic “polycrisis” characterized by political instability, energy shortages, ecological disruption, and the catastrophic 2022 floods that caused an estimated $14.9 billion in damages and severely impeded economic recovery [7–10]. As these overlapping crises intensified material insecurity and uncertainty, economic and environmental vulnerabilities became increasingly intertwined. Thus, the persistent and compounded economic strains represent a direct extension of the initial COVID-19 recession, situating Pakistan firmly within a prolonged post-COVID-19 recessionary context.

Within such prolonged recessionary conditions, intersecting economic and ecological crises can substantially reshape environmental priorities and behaviors [11, 12]. Economic conditions influence public perceptions of climate change and sustainability [13]. In some cases, economic crises may encourage more resource-conscious and sustainable practices through heightened perceptions of scarcity and personal responsibility [14, 15]. Conversely, periods of financial instability may also narrow individuals’ temporal and moral horizons, shifting attention away from long-term environmental concerns toward immediate material needs [16]. Despite growing research on pro-environmental behavior, limited attention has been given to how prolonged macroeconomic crises shape environmental decision-making through affective and cognitive responses, particularly among younger generations navigating sustained economic uncertainty.

Existing scholarship on pro-environmental behavior has primarily emphasized relatively stable psychological and socio-demographic predictors, including environmental concern, perceived behavioral control, personal norms, attitudes, gender, and education [17–21]. More recent studies have also explored the relationship between environmental behavior and economic outcomes within organizational settings, demonstrating how workplace-oriented pro-environmental actions can contribute to operational efficiency and economic performance [22, 23]. Together, these strands of research have substantially advanced understanding of the economic-environment linkage. However, environmental behavior remains largely conceptualized within psychological, socio-demographic, and organizational structures. Thus leaving environmental decision-making comparatively underexplored within contexts of prolonged macroeconomic disruption, particularly in relation to how economic crises shape affective and cognitive responses at the individual level. Existing research further suggests that perceived lack of control and uncertainty regarding environmental outcomes can produce forms of “environmental numbness,” in which environmental issues are experienced as distant or psychologically abstract [24–26]. However, the convergence of post-pandemic inflation and escalating ecological disasters, including the catastrophic 2022 floods in Pakistan, has rendered environmental and economic vulnerabilities increasingly immediate and materially consequential. In such contexts, understanding how individuals cognitively and affectively respond to economic crises becomes critical for examining the conditions under which pro-environmental behavior may either intensify or recede.

Among the populations most significantly shaped by these prolonged conditions of uncertainty and instability is Generation Z (referred to as “Gen-Z”). Born between 1995 and 2010 [27], Gen-Z has navigated critical developmental stages during the political-unrest, global financial crises, and recently, the COVID-19 pandemic. Contemporary evidence suggests that Gen-Z exhibit enhanced awareness and concerns about environmental conservation, as compared to earlier generations [28, 29]. Moreover, Gen-Z is regarded as the largest generation, comprising 32% of the world’s population and 30% of Pakistan’s population. It is also projected to become the largest group of active consumers by 2030 [30, 31]. As consumptions patterns in future will be influenced by evolving preferences of newer generations [32], thus, the study of Gen-Z’s pro-environmental behavior during economic recessions represents an important yet understudied area. While previous studies have examined consumer responses to economic downturns [33, 34], there remains limited historical precedent for economic crises coinciding with the formative years of Generation Z. Combined with this cohort’s distinct digital connectivity and heightened environmental consciousness [35, 36], this context underscores the need for further empirical research to elucidate the interplay between economic conditions and environmental behaviors among this demographic.

Affective Events Theory (AET) offers a framework to link external events and their cognition and describes how specific events impact individual decision-making behaviors [37]. AET contrasts with the more commonly used theory of planned behavior [38], instead examining the influence of affective-reactions triggered by work events on employees’ behaviors [39]. Although originally developed within organizational settings to examine how workplace events influence employee behavior, the broader emphasis of AET on affective appraisal processes provides analytical flexibility for examining emotionally consequential disruptions beyond workplace environments. AET thus suggests that emotionally charged events can induce both positive and negative affective reactions that can substantially impact individual’s motivations and behavior. While existing AET research has concentrated on internal work events and public health events [40, 41], the role of prolonged macro-level disruptions, particularly economic recessions, in shaping pro-environmental behavior through affective pathways remains comparatively underexplored.

In light of AET, as behaviors can stem directly from affective reactions [39], the cognition of economic recession can potentially either enhance the pro-environmental behaviors of Gen-Z, given their strong apparent commitment to socio-ecological issues [42], or it may also potentially overshadow their prioritization of pro-environmental actions because of the stress to establish economic-stability, given the critical stage in their lives where they navigate the complexities of education, workforce entry, and financial independence.

To scrutinize these potentially profound yet understudied effects of Post-Covid-19 economic recession cognition on Gen-Z’s environmental decision making, the present research develops and empirically tests an integrative model examining how cognition of the post-COVID-19 economic recession shapes positive and negative environmental affective reactions in Gen-Z, and how these reactions subsequently influence pro-environmental behaviors across household, public, workplace, and financial spheres. Understanding these dynamics is particularly important as economic crises increasingly intersect with environmental challenges, shaping how younger generations prioritize sustainability during periods of financial uncertainty.

Conceptually, the study extends the scope of AET beyond organizational and public-health related events to encompass economic recessions and emergencies. In doing so, it complements organizational-level research linking environmental behavior and economic outcomes [22]. Moreover, situated within the context of a post-COVID-19 economic recession, the study links individuals’ cognition of economic downturns to a range of pro-environmental behaviors. A central innovation of the study is the introduction and empirical examination of financial-sphere PEB as a distinct domain of action through which economic stress may reshape environmental decision-making and youth financial agency, something that remains underexplored in behavioral models and policy discourse [43, 44]. By integrating economic crisis cognition, affective responses, and multi-sphere environmental behaviors, the study contributes an analytical basis for understanding how crises recalibrate priorities across different domains of action. In doing so, this research offers decision-relevant insights for planners and policymakers, positioning economic disruptions as opportunities to embed sustainability into recovery strategies and build more durable socio-economic and environmental resilience.

## Literature review & research model development

### Gen-Z and the green paradox

Generational theory identifies a generation as individuals born within a particular time frame, sharing socio-cultural and historical experiences that shape their behaviors and values [45]. Applying this framework, Generation Z, also referred as the “iGeneration,” has been profoundly influenced by early exposure to the internet and social media [46], making them technologically adept and characterized by diverse social values and a keen awareness of global affairs, especially environmental issues.

Extant literature underscores Gen Z’s pronounced eco-consciousness relative to previous generations. Corner et al. [47] revealed that Gen Z exhibits heightened environmental concern, stemming from an acute sense of urgency about climate change. Similarly, Agrawal et al. [48] linked their exposure to constant information streams on social media to increased environmental awareness. Further, studies by Calista and Yenni [49] and Haugestad [50] highlight Gen Z’s active engagement with environmental issues, exemplified by their leadership in movements like Fridays for Future climate strikes.

Nonetheless, this environmental concern does not always translate into consistent Pro-environmental Behaviors (PEBs). Casalegno et al. [51] found that, despite strong environmental values, Gen Z is less likely to act on them, such as by purchasing green products, compared to older generations. Gifford [52] attributed this discrepancy to barriers like convenience and lack of immediate incentives. Otto & Pensini [53] also observed this paradox, noting that while Gen Z champions environmental policies, their personal PEBs are less habitual than those of older generations. In Pakistan, where Gen Z constitutes about 30% of the population [30], studies on their environmental behaviors are limited. Existing research indicates that while Gen Z shows interest in green products, financial constraints hinder consistent PEBs [54].

Cumulatively, the literature presented above indicate that economic conditions are not merely background barriers but may actively shape whether environmental concern is translated into behavior. However, existing studies have largely examined financial constraints as individual-level impediments, while giving less attention to how broader recessionary contexts influence young people’s environmental meaning-making, emotional responses, and behavioral choices. The post-COVID-19 economic recession therefore provides a critical context for examining whether economic hardship amplifies or narrows Gen-Z’s concern-action gap, and through what affective processes this may occur. To examine these affective processes more directly, the current study draws on Affective Events Theory (AET), which provides a framework for understanding how cognitively appraised events generate emotional responses that subsequently influence behavior.

### Post-COVID-19 economic recession cognition, affective events theory (AET) and environmental affective reactions

Unprecedented economic emergencies, pose multifaceted threats to human well-being, potentially encouraging or discouraging sustainable behaviors [12]. Several theoretical models, including the Theory of Planned Behavior [38], Value-Belief-Norm Theory [55], and Norm Activation Model [56] have been employed to investigate environmental behavior mechanisms. Recent sustainability research has further emphasized the role of contextual and motivational pathways in shaping environmentally oriented behavior [57]. However, these models often emphasize relatively stable attitudes, values, norms, or perceived control, whereas the present study is concerned with how an economically disruptive event is cognitively appraised and affectively translated into behavior. In this sense, Affective Events Theory (AET) offers a fresh perspective by focusing on how individuals’ emotional reactions to event cognition influence subsequent behaviors [37]. Although originally developed in organizational settings, the central mechanism of AET is not limited to workplaces; rather, it concerns the affective appraisal of events that are experienced as consequential for individuals’ goals, resources, and well-being. This way, prolonged recessionary conditions can be understood as macro-level affective events when they are lived through financial insecurity, uncertainty, and constrained coping capacity. This study therefore applies AET to examine how Gen-Z’s cognition of the post-COVID-19 recession shapes positive and negative environmental affective reactions and, subsequently, pro-environmental behaviors. It is important to acknowledge, however, that AET, as explained earlier, was originally developed to explain affective responses to discrete workplace events rather than prolonged societal crises. Economic recessions differ from organizational events in their duration, scale, and indirect modes of exposure, meaning that affective responses may also be shaped by broader structural and contextual influences. Nevertheless, the core premise of AET, that cognitively appraised events generate affective reactions that subsequently influence behavior, remains relevant for examining how individuals experience and respond to prolonged recessionary conditions.

AET suggests that behavioral responses do not emerge directly from external conditions alone, but from how such conditions are cognitively interpreted and affectively experienced [38]. Under prolonged recessionary conditions, individuals may differ considerably in how they perceive the severity, personal relevance, and manageability of economic disruption. For some, heightened perceptions of emergency and vulnerability may intensify concern toward broader socio-ecological crises and encourage more resource-conscious behaviors. For others, persistent financial insecurity may narrow behavioral priorities toward immediate coping and survival-oriented concerns. In this regard, Lazarus [58] conceptualizes emergency cognition as comprising Emergency Relevance (ER) and Emergency Coping (EC). ER, or primary appraisal, reflects the extent individuals perceive crises as critical to personal well-being, while EC, or secondary appraisal, pertains perceived resources and adaptability. In the recessionary context, these constructs jointly serve as antecedents, triggering diverse affective reactions that mediate the relationship between emergency cognition and behavior.

Clore et al. [59] define affective reactions as emotional responses to external environment, and can be either positive or negative. Positive environmental affective reactions include emotions like pride and hope, arising from sustainable behaviors, while negative reactions such as anxiety and guilt emerge from awareness of ecological degradation [60]. Economic recessions can amplify both reactions. Under recessionary conditions, both forms of affective reaction may intensify. For instance, resource-conscious and cost-saving sustainable practices may generate feelings of pride and efficacy, while financial constraints and reduced behavioral flexibility may simultaneously provoke guilt, frustration, or anxiety regarding unsustainable practices [61].

Recession may intensify the intersection between economic insecurity and environmental concern. Particularly, as climate change intensifies ecological disasters [62], their long-term economic repercussions complicate recovery efforts [63], deepening the intersection between these crises [64]. In this context, Gen-Z’s Emergency Relevance and Emergency Coping assessments likely influence their affective reactions. Prior empirical research indicates that emotionally charged contexts can intensify environmental emotions such as guilt, anxiety, and pride, which subsequently influence environmental behaviors and engagement [65, 66]. For Gen Z navigating financial uncertainty alongside heightened awareness of socio-ecological crises, recession cognition may therefore activate both positive and negative environmental affective reactions. Accordingly, the present study proposes the following hypotheses:

#### **H1**

Cognition of post-COVID-19 recession exerts an increasing influence on positive environmental affective reactions (PEAR).

- **H1a**: Perceived emergency relevance regarding the post-COVID-19 recession has a positive effect on PEAR.
- **H1b**: Perceived emergency coping regarding the post-COVID-19 recession has a positive effect on PEAR.

#### **H2**

Cognition of Post-COVID-19 recession exerts a positive influence on negative environmental affective reactions (NEAR).

- **H2a**: Perceived emergency relevance regarding the post-COVID-19 recession has a positive effect on NEAR.
- **H2b**: Perceived emergency coping regarding the post-COVID-19 recession has a positive effect on NEAR.

### Environmental affective reactions and pro-environmental behaviors

While the preceding section established how recession cognition may shape positive and negative environmental affective reactions, AET further suggests that these affective responses become consequential insofar as they influence subsequent behavioral choices. The following section therefore examines how environmental affective reactions may translate into pro-environmental behavior across different spheres of action. Kollmuss and Agyeman [67] define Pro-environmental Behaviors (PEBs) as purposeful actions aimed at reducing environmental harm. Various scholars have explored different dimensions of PEB [68, 69]. Mi et al. [70] recently categorized PEB intentions into three spheres based on their field of occurrence: household, workplace, and public spheres. Our study adopts these three spheres while expanding them to suit the focus on Gen-Z. The household sphere includes everyday domestic actions like energy conservation and water management [71]. The work sphere encompasses behaviors promoting environmental sustainability in professional settings, such as resource efficiency [72]. For Gen-Z, this study extends the work-sphere to include educational institutions, where many of them spend significant time. PEB in educational settings may involve participation in campus sustainability initiatives and efforts to reduce paper usage, with studies like Zsóka et al. [73] highlighting such engagement as a key predictor of students PEBs. Finally, the public sphere includes activities that influence broader societal outcomes, such as community clean-ups or participation in environmental protests [68].

Yet financial behaviors increasingly constitute an important site through which environmental commitments are expressed, negotiated, and constrained, particularly under conditions of economic uncertainty. This is especially relevant for Gen-Z, whose growing demographic and future economic influence contrasts with persistent assumptions that younger populations possess limited financial agency [82, 83]. Accordingly, the financial sphere in this study encompasses a broader range of environmentally relevant financial behaviors, including support for green companies, sustainable investment preferences, and advocacy for environmentally responsible economic practices [84, 85]. Such behaviors become particularly significant during recessionary conditions, where financial commitment toward environmental protection may become simultaneously more difficult and more consequential [86]. Prior research further suggests that environmental emotions such as anticipated pride and guilt may shape environmentally oriented consumption decisions and broader financial choices [87, 88], indicating that affective reactions may also influence environmental behavior within the financial sphere.

In addition, this study extends Mi et al.‘s [70] model by conceptualizing a financial sphere for PEB, addressing a gap in existing literature that often overlooks financial behaviors. Financial contributions, such as supporting green companies or investing in sustainable funds, are critical yet underexplored as pointed by Waheed and Waheed [74]. For instance, a substantial body of research scrutinized willingness to pay (WTP) for eco-friendly products as a measure of PEB. García-Salirrosas et al. [75] found that consumer’s WTP is influenced by environmental labeling, while Moser [76] demonstrated that WTP is a predictor of purchasing green products. Similarly, Fairbrother [77] and Saunila [78] have explored variables including investment in green technologies. However, these variables often fail to encapsulate the broader range of financial behaviors related to PEBs. In contemporary times, characterized by environmental awareness and economic uncertainties, it is critical to recognize the financial-sphere as a separate domain within PEB. This is especially relevant for Gen-Z as existing behavioral models often overlook youth’s financial spheres, largely due to the presumption that young people lack the capacity or agency to manage significant financial matters, a view that neglects their growing demographic and future economic influence [79]. Recognizing this gap is imperative, particularly as Generation Z emerges as a dominant population cohort whose financial behaviors and needs deserve systematic consideration [80]. This sphere extends beyond just WTP and includes a variety of financial behaviors, including support of green companies, investment in sustainable funds, and advocating for incentivization of sustainable energy [81, 82]. As individual’s financial commitment to environmental protection becomes important yet difficult during times of crisis [83]. Prior studies suggest that self-conscious environmental emotions are not confined to symbolic concern alone but can shape consumption-related decisions too, with anticipated pride and guilt influencing intentions to buy sustainable products and broader pro-environmental decision making [84, 85]. These actions reflect a financial commitment to environmental sustainability, especially significant in the context of an economic recession.

During recessions, individual’s affective reactions can influence PEB in complex and sometimes contradictory ways. As Shum’s [86] study indicates, economic anxiety may prioritize personal benefit over conservation. Conversely studies like Waheed et al. [12] show that resource scarcity drives sustainability as individuals lack means to counter climate change’s impacts. As Pakistan’s current recession is also marked by several ecological crises such as Winter Smog, floods and heat waves [87, 88], and during such times, the Pakistani government activated several public emergency responses including public awareness campaigns and behavioral emergency interventions [89], which potentially can inspire public to engage more in sustainable behaviors [90]. Thus the simultaneous interplay of both, positive and negative affective reactions during current economic recession can directly influence PEB and give new insights into the PEB that have not been explored before. Additionally, for Gen-Z, a cohort characterized by increased environmental awareness but limited financial agency, understanding influence of economic recession on their PEBs, particularly within the financial-sphere, is crucial.

Previous empirical research provides a basis for expecting both positive and negative environmental affective reactions to shape pro-environmental engagement. Studies on young people’s climate engagement show that constructive hope can function as a motivational force for pro-environmental behavior [91]. Concurrently, work on anticipated self-conscious emotions suggests that pride and guilt can operate as self-regulatory mechanisms in environmentally relevant decision-making, including intentions to purchase environmentally friendly products and broader pro-environmental choices [84, 85]. At the same time, evidence also suggests asymmetry in these effects, with pride often showing a more consistent positive association with subsequent pro-environmental action, while guilt may be more contingent on contextual factors such as perceived norms and opportunities for action [92]. In aggregate, this literature supports the expectation that both positive and negative environmental affective reactions may influence pro-environmental behavior, including in contexts where economic strain reshapes the feasibility and salience of action across different spheres. Based on these theoretical arguments and the supporting empirical evidence, we hypothesize:

#### **H3**

Positive environmental affective reactions positively influence Pro-environmental Behavior (PEB).

- **H3a**: Positive environmental affective reactions regarding the post-COVID-19 recession positively influence household-sphere PEB.
- **H3b**: Positive environmental affective reactions regarding the post-COVID-19 recession positively influence work-sphere PEB.
- **H3c**: Positive environmental affective reactions regarding the post-COVID-19 recession positively influence public-sphere PEB.
- **H3d**: Positive environmental affective reactions regarding the post-COVID-19 recession positively influence financial-sphere PEB.

#### **H4**

Negative environmental affective reactions positively influence Pro-environmental Behavior (PEB).

- **H4a**: Negative environmental affective reactions regarding the post-COVID-19 recession positively influence household-sphere PEB.
- **H4b**: Negative environmental affective reactions regarding the post-COVID-19 recession positively influence work-sphere PEB.
- **H4c**: Negative environmental affective reactions regarding the post-COVID-19 recession positively influence public-sphere PEB.
- **H4d**: Negative environmental affective reactions regarding the post-COVID-19 recession positively influence financial-sphere PEB.

**Fig. 1:**
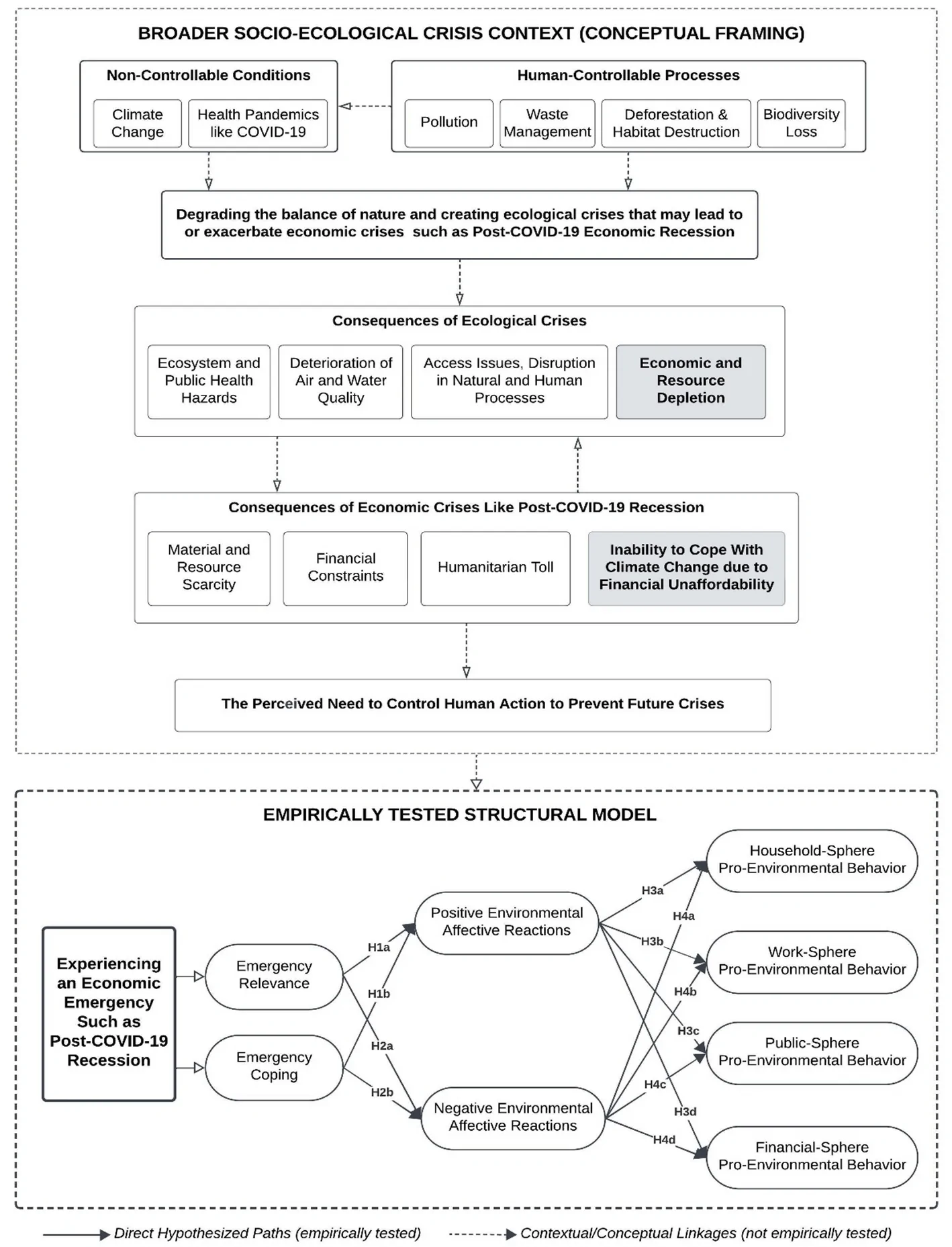
Conceptual Socio-Ecological Crisis Context Informing the Empirically Tested Affective Pathways Model

Based on the preceding discussion, the conceptual framework (Fig. 1) illustrates the broader socio-ecological crisis context conceptually informing the study and the empirically tested structural relationships examined through Structural Equation Model (SEM) analysis. The upper section contextualizes how ecological disruptions, economic crises, and broader socio-environmental conditions may shape environmental meaning-making and perceptions surrounding the post-COVID-19 recession. Informed by the broader contextual framing, the empirically tested model examines how Gen-Z’s cognition of recessionary conditions, through Emergency Relevance and Emergency Coping appraisals, may influence positive and negative environmental affective reactions and subsequently shape pro-environmental behavior across household, work, public, and financial spheres.

## Research methodology

### Data collection and sampling

We used a cross-sectional research design targeting Gen-Z individuals i.e. those born between 1995 and 2010 [27]. A quantitative survey (*N* = 352) was conducted between June and July 2024 following a pilot (*N* = 70) from the target population, where pre-testing yielded Cronbach’s alphas exceeding 0.70 for all constructs indicating strong reliability [93]. From 352 responses collected, 32 incomplete responses were deleted for a completion rate of 90.9%.

The inclusion criteria for our target population was Gen-Z membership according to age, and spatial location across the federal capital and all 5 provincial capital cities of Pakistan; Islamabad (and Rawalpindi), Karachi, Lahore, Peshawar, Quetta and Gilgit, through an active nationwide youth network of Pakistan, known for its extensive engagement with Gen-Z individuals. Although the data was collected from urban centers, the methodology ensured the rural members inclusion within the network, thereby improving demographic and geographical diversity within the sample.

Firstly, a purposive sampling framework was used to deliberately recruit respondents with different levels of personal environmental awareness. The sampling strategy did not pre-screen respondents according to environmental concern. Instead, recruitment was diversified across educational institutions, youth organizations, social networks, and regional contacts, rather than being limited to environmental groups or climate-focused organizations. This approach was intended to broaden the recruitment base beyond already environmentally active young people. Environmental concern and related orientations were then measured within the survey and incorporated analytically, rather than used as recruitment quotas. Nevertheless, because participation was voluntary, the possibility of self-selection among environmentally interested respondents remains a limitation and should be considered when interpreting the generalizability of the findings. Secondly, the sampling was then extended via snowballing of respondents, where selected respondents recommended others fitting the inclusion criteria. Although snowballing can potentially limit generalizability, it also offers the benefit of accessing hard-to-reach populations making it suitable for our study [94].

Sample size adequacy was evaluated using multiple benchmarks. First, a priori power analysis was conducted with G*Power 3.1 [95]. Assuming a medium effect size (f^2^=0.15), a maximum of two predictors, an alpha level of 0.05, and desired statistical power of 0.80, the required minimum sample size was estimated to be approximately 70 participants. Second, we applied 10:1 rule, which recommends a minimum ratio of ten participants for every estimated parameter or indicator, as outlined by Kline [96] and cited widely in the literature [97]. Our final sample of 320 participants satisfied both criteria, thereby providing a sufficient sample size, ensuring both statistical power and adequacy for conducting Partial Least Squares Structural Equation Modeling (PLS-SEM) [98]. All the data gathered from respondents supporting the replicability of this research is available at doi:https://doi.org/10.17632/vmft4d24h6.1 [99]. Ethical standards ensured integrity, consent, confidentiality, anonymity, and secure data storage (University of Leeds Ethical Approval# AREA-20-070). The socio-demographic characteristics of the sample are given in Table 1.

**Table 1:** Socio-demographic Characters of the Sample

Socio-demographics	Categories	Number	Percentage of Sample %
Gender	Male	152	47.5
	Female	161	50.3
	Non-binary/ Transgender	7	2.2
Age	16 to 20	81	25.3
	21 to 24	144	45.0
	25 to 29	95	29.7
Education Level	Below Junior High School	44	13.8
	High School or Secondary School Degree	58	18.1
	Bachelor’s Degree	108	33.8
	Graduate Degree or Above	110	34.4
Profession	Student	139	43.4
	Employed	116	36.3
	Self-Employed	39	12.2
	Unemployed	9	2.8
	Housewife or Other	17	5.3
Monthly Household Income	Below 30,000 PKR	95	29.7
	31,000 to 99,000 PKR	124	38.8
	100,000 PKR to 299,000 PKR	77	24.1
	Above 300,000 PKR	24	7.5

### Measures

The study adapted validated scales from the literature. Minor contextual and wording modifications were introduced to improve situational relevance for Pakistani Gen-Z respondents experiencing post-COVID-19 economic recession conditions, while maintaining conceptual consistency with the original constructs. Following prior recommendations, such as those of Sarwar et al. [100], regarding instrument adaptation and contextual validity, a pilot study (*n* = 70) was conducted prior to the main survey to assess clarity, reliability, and contextual appropriateness of the measures. The questionnaire comprised of 32 items and eight constructs, as given in Table 2 (Complete Questionnaire attached as supplementary material). All measures were rated on a 5-point Likert scale ranging from 1 (strongly disagree) to 5 (strongly agree) except for variables measuring self-reported pro-environmental behaviors in all four spheres which ranged from 1 (rarely) to 5 (always).

**Table 2:** Construct Reliability and Convergent Validity

Constructs		Items		Std. Loadings		Cronbach’s α		AVE		CR	
		ER_1		0.83							
		ER_2		0.79							
Emergency Relevance (ER)		ER_3		0.70		**0.781**		0.60		0.85	
		ER_4		0.77							
		EC_1		0.74							
		EC_2		0.62							
Emergency Coping (EC)		EC_3		0.83		**0.726**		0.54		0.82	
		EC_4		0.72							
		PEAR_1		0.74							
Positive Environmental Affective Reactions (PEAR)		PEAR_2		0.75							
		PEAR_3		0.80		**0.817**		0.63		0.87	
		PEAR_4		0.88							
		NEAR_1		0.77							
Negative Environmental Affective Reactions (NEAR)		NEAR_2		0.77							
		NEAR_3		0.74		**0.758**		0.57		0.84	
		NEAR_4		0.74							
		HS-PEB 1		0.81							
Household Sphere Pro-Environmental Behaviors (HS-PEB)		HS-PEB 2		0.82							
		HS-PEB 3		0.83		**0.840**		0.67		0.89	
		HS-PEB 4		0.80							
		WS-PEB 1		0.71							
		WS-PEB 2		0.78							
Work Sphere Pro-Environmental Behaviors (WS- PEB)		WS-PEB 3		0.83							
		WS-PEB 4		0.79		**0.794**		0.61		0.86	
Public Sphere Pro-Environmental Behaviors (PS-PEB)		PS-PEB 1		0.71							
		PS-PEB 2		0.87							
		PS-PEB 3		0.85		**0.824**		0.65		0.88	
		PS-PEB 4		0.78							
		FS-PEB 1		0.76							
Financial Sphere Pro-Environmental Behaviors (FS-PEB)		FS-PEB 2		0.74							
		FS-PEB 3		0.83		**0.779**		0.59		0.85	
		FS-PEB 4		0.74							

AVE= average variance extracted; CR=composite reliability

#### Economic recession emergency cognition

Economic recession emergency cognition was measured using an adapted version of Folkman et al.’s scale [101], consisting of two dimensions: emergency relevance and emergency coping. Items were contextually modified to reflect post-COVID-19 economic recession conditions in Pakistan while retaining the conceptual foundations of the original appraisal framework. Both subscales comprised 4-items each.

#### Positive and negative environmental affective reactions

To measure environmental affective reactions, scales developed by Lazarus [102, 103] were adapted to capture environmentally oriented affective reactions emerging within post-COVID economic recession conditions. Both the positive and negative environmental affective reactions scale comprised of 4-items each.

#### Pro-environmental behaviors

We evaluated Gen-Z’ PEB across four spheres; household, public, work, and financial spheres. Accordingly, four relevant validated scales were adopted for each sphere and included 4-items per sphere. For the household and public-sphere PEBs, two dimensions of the scale developed by Mateer et al. [104], were adopted to the Pakistani context. The work-sphere PEB was measured using the scale by Bissing-Olson et al. [105]. Given that the current research population comprised of Gen-Z individuals, both in work and education, the scale was adapted to encompass both workplace and educational contexts to capture the diverse behaviors of Gen-Z participants in various settings. Finally, financial-sphere PEB was measured using Chan’s [106] scale assessing environmentally oriented financial support and decision-making tendencies. The construct was retained in the present study because environmentally consequential financial orientations, including support for sustainable products, environmentally responsible organizations, and green financial preferences, represent an increasingly important dimension of pro-environmental engagement, particularly under conditions of economic uncertainty and has been used in previous studies such as Eom et al. [107].

### Analytical strategy

This study’s data analysis is divided into three sections. Firstly, a measurement model was built to ensure the reliability and discriminant validity of all constructs [98]. Secondly, a structural model was scrutinized, using the metrics discussed in Thakkar & Thakkar [108]; coefficient of determination (R^2^), effect size (f^2^), predictive relevance (Q^2^), and overall goodness-of-fit. Finally, a bootstrapping approach with a 95% confidence interval was utilized for hypothesis testing. The suggested model and accompanying hypotheses (Fig. 1) were evaluated using Partial Least Squares Structural Equation Modelling (PLS-SEM). We used PLS-SEM to measure the effect of latent variables while controlling for potential measurement error. It is an improvement on multiple regression in this context and has been widely used to jointly explain multiple environmental factors [109]. The research employed SmartPLS software Version 4.1.0.6 for analysis [110].

### Common methods bias

To avoid common method bias, both a priori and post-hoc remedies were applied as suggested by Podsakoff et al. [111]. For a priori measures, the questionnaire included socio-demographic questions between construct scales. This design distinguished between the measurement of independent and dependent variables, therefore reducing the likelihood that consistent responses were due to the measurement method rather than the actual constructs being measured, as recommended by Jordan & Troth [112].

Exploratory factor analysis using Harman’s single factor test was conducted as a post-hoc measure [113]. The highest variance explained by a single un-rotated factor was 20.84%, well below the recommended 50% threshold, suggesting that common method variance bias was absent. However, this method may be limited due to reliance on a single-factor [114, 115]. Therefore, the marker variable approach [116] was also applied to address common method bias, for which two separate Partial-Least-Squares (PLS) structural models were estimated: one including the marker variable as an exogenous predictor for every construct, and the other model excluding the marker variable. The results indicated insignificant effects of marker variable on the endogenous constructs. Additionally, the results reported no significant differences in the outcomes between the two PLS models, concluding that common methods bias did not present a significant threat to this data.

## Results

### Measurement model

We validated the PLS-SEM modelling using Cronbach’s alpha (CA), factor loadings, composite reliability (CR), and average variance extracted (AVE) as recommended by Hair et al. [117]. The results in Table 2 indicate that for all constructs the values of Cronbach’s alpha, rho_A, and composite reliability (CR) exceeded the critical value of 0.70 and ranged from 0.72 to 0.89. The factor loadings were above the suggested minimum of 0.50 and ranged from 0.62 to 0.88. Moreover, average variance extracted (AVE) values for all constructs were greater than the recommended threshold of 0.50 and ranged from 0.54 to 0.67 [117]. Thus, the result values confirmed that the measurement model possessed strong internal consistency and convergent validity.

For testing the discriminant validity of the measurement model, two methods i.e. Fornell and Larcker criterion [118] and the heterotrait–monotrait (HTMT) ratio [119] were employed. For every construct, the AVE values exceeded the highest correlation values observed with other constructs, thereby satisfying the Fornell and Larcker criterion. As for the Heterotrait-Monotrait Ratio (HTMT), the values were below the threshold of 0.85, further validating the discriminant validity of the measurement model. The results for discriminant validity are shown in Table 3.

**Table 3:** Means, Standard deviations and Discriminant Validities with bold diagonal-elements showing square root of AVE, values below them showing the Fornell–Larcker Criterion, values above diagonal present HTMT-ratio

	Mean	SD	1	2	3	4	5	6	7	8
Emergency Relevance	3.836	0.762	0.777	0.089	0.394	0.326	0.528	0.280	0.388	0.385
Emergency Coping	3.586	0.667	0.018	**0.736**	0.318	0.306	0.103	0.247	0.217	0.239
Positive Environmental Affective Reactions	2.939	0.838	0.338	−0.246	**0.799**	0.075	0.213	0.231	0.261	0.161
Negative Environmental Affective Reactions	3.903	0.606	0.255	0.249	0.034	**0.760**	0.390	0.423	0.224	0.441
Household-sphere Pro-environmental Behavior	3.707	0.783	0.425	0.067	0.196	0.317	**0.822**	0.355	0.314	0.359
Work-sphere Pro-environmental Behavior	3.407	0.774	0.222	0.159	0.191	0.334	0.294	**0.784**	0.700	0.775
Public-sphere Pro-environmental Behavior	2.902	0.907	0.309	0.133	0.240	0.188	0.265	0.566	**0.808**	0.790
Financial-sphere Pro-environmental Behavior	3.434	0.813	0.296	0.176	0.132	0.351	0.292	0.600	0.605	**0.773**

### Reflective model validation

To enhance the accuracy of the measurement model and further ensure parameter accuracy, a Confirmatory Tetrad Analysis (CTA-PLS) was conducted adhering to Gudergan et al. [120]’s guidelines. This analysis aimed to identify any misspecifications related to reflective constructs in the measurement model. The results revealed the tetrad value for each construct to be zero, confirming that all the constructs were measured reflectively, validating the robustness of the reflective measurement.

### Structural model

To assess the structural model, multiple metrics were calculated, including effect size (f²), R-square (R²), Q-square (Q²), Variance Inflation Factor (VIF), predictive relevance, and multicollinearity [121]. Effect size (f²) values, presented in Table 5, ranged from 0.022 to 0.143, all exceeding the threshold of 0.020, which indicates a true effect [117]. Thus, the model demonstrated a strong intensity of association between the dependent and independent variables.

R² values, representing the variance in the dependent variable explained by the independent variables, ranged from 0.09 to 0.18. While modest, these values align with prior research, where R² values below 0.20 are typical in studies involving human behavior due to its inherent complexity [121]. Significant path coefficients (*p* < 0.05, detailed in Sect. 4.4) further substantiate that the independent variables meaningfully predict the dependent variables.

Employing Stone-Geisser’s Q², predictive relevance was assessed, yielding values from 0.055 to 0.157. Since all Q² values exceeded zero, the model demonstrated substantial predictive relevance for the constructs, as per [117]. Additionally, VIF values, ranging from 1.469 to 2.175, were below the common threshold of 5 [121], validating that multicollinearity among the constructs was not an issue. Detailed values of R², Adj R², Q² and VIF are presented in Table 4.

To complete model evaluation, the Standardized Root Mean Square Residual (SRMR) method was employed to assess overall fit. Preferred for smaller, complex models, SRMR evaluates model fit more effectively than criteria like CFI or RMSEA [122]. The SRMR value of 0.070 fell below the accepted threshold of 0.080, as recommended by Henseler et al. [119], confirming the model as a good fit. Thus, the model satisfied all criteria for evaluation.

**Table 4:** Structural Model Analysis

Paths	R ^2^	Adj. R^2^	Q^2^	VIF
Emergency Relevance	–	–	–	1.569
Emergency Coping	–	–	–	1.718
Positive Environmental Affective Reactions	0.178	0.173	0.157	2.108
Negative Environmental Affective Reactions	0.124	0.119	0.103	1.469
Household-sphere Pro-environmental Behavior	0.135	0.129	0.098	1.968
Work-sphere Pro-environmental Behavior	0.144	0.139	0.050	2.175
Public-sphere Pro-environmental Behavior	0.090	0.084	0.055	1.683
Financial-sphere Pro-environmental Behavior	0.138	0.132	0.070	1.726

### Hypothesis testing

Table 5 shows that perceived Emergency Relevance (ER) was significantly associated with Positive Environmental Affective Reactions (β = 0.343, *p* < 0.001, supporting H1a) and Negative Environmental Affective Reactions (β = 0.250, *p* < 0.001, supporting H2a). Perceived Emergency Coping (EC) reduced Positive Environmental Affective Reactions (β = −0.252, *p* < 0.001), contrary to the hypothesized direction of H1b, while increasing Negative Environmental Affective Reactions (β = 0.244, *p* < 0.001, supporting H2b).

The direction of these relationships matters because PEAR positively affected PEBs across household-sphere (β = 0.186, *p* < 0.01), work-sphere (β = 0.180, *p* < 0.01), public-sphere (β = 0.234, *p* < 0.001), and financial-sphere behaviors (β = 0.120, *p* < 0.05). NEAR also positively influenced household-sphere (β = 0.310, *p* < 0.001), work-sphere (β = 0.328, *p* < 0.001), public-sphere (β = 0.347, *p* < 0.001), and financial-sphere behaviors (β = 0.180, *p* < 0.001). Together, all hypotheses were supported except for H1b, as EC demonstrated a significant but inverse relationship with PEAR. Further, we found that PEBs in all four spheres were more strongly affected by negative affective reactions rather than positive.

**Table 5:** Results of Structural Model Hypothesis Testing

Paths		Effects	SE	f^2^	t-value	*p*-values	Hypothesis Results
		*Emergency Relevance* →* Environmental Affective Reactions*					
H1a: ER → PEAR		0.343***	0.059	0.143	5.844	0.000	Supported
H2a: ER →NEAR		0.250***	0.056	0.071	4.459	0.000	Supported
		*Emergency Coping* →* Environmental Affective Reactions*					
H1b: EC → PEAR		-0.252***	0.058	0.077	4.387	0.000	Not Supported (due to the negative effect)
H2b: EC → NEAR		0.244***	0.059	0.068	4.151	0.000	Supported
		*Positive Environmental Affective Reactions*→* PEBs*					
H3a: PEAR → HS-PEB		0.186**	0.058	0.040	3.207	0.001	Supported
H3b: PEAR →WS-PEB		0.180**	0.057	0.038	3.145	0.002	Supported
H3c: PEAR → PS-PEB		0.234***	0.063	0.060	3.721	0.000	Supported
H3d: PEAR → FS-PEB		0.120*	0.059	0.022	2.019	0.044	Supported
		*Negative Environmental Affective Reactions*→* PEBs*					
H4a: NEAR → HS-PEB		0.310***	0.063	0.111	4.898	0.000	Supported
H4b: NEAR → WS-PEB		0.328***	0.043	0.125	7.607	0.000	Supported
H4c: NEAR → PS-PEB		0.347***	0.048	0.036	7.257	0.000	Supported
H4d: NEAR → FS-PEB		0.180***	0.050	0.140	3.594	0.000	Supported

ER = emergency relevance; EC = emergency coping; PEAR = positive environmental affective reactions; NEAR = negative environmental affective reactions; HS-PEB = household-sphere PEB; WS-PEB = work-sphere PEB; PS-PEB = public-sphere PEB; FS-PEB= financial-sphere PEB; SE= standardization error.* *p <* 0.05, **p < 0.01, ***p < 0.001.

As affective reactions can mediate the association between the cognition of post-COVID-19 economic recession and PEBs, we subsequently scrutinized the potential indirect effects by employing a bootstrapping method with 10,000 subsamples at a 95% confidence interval (CI) and 5% significance level (two-tailed) as recommended by Preacher & Hayes [123].

ER demonstrated significant indirect effects on most spheres of PEB through both positive and negative affective reactions (Table 6). Although ER, through positive affect, showed a significantly positive indirect effect on household-sphere, work-sphere, and public-sphere PEBs, it did not statistically influence the financial-sphere PEB (Path Coefficient = 0.041, *p* = 0.085), suggesting that financial pro-environmental behaviors might be less influenced by positive environmental reactions in the context of ER.

In contrast, the role of EC portrays a more complex picture. EC demonstrated significant indirect effects on PEBs across all spheres through NEAR, indicating that EC may indirectly contribute to PEBs through negative affective reactions. Contrastingly, through PEAR, EC demonstrated a significant but negative indirect effect on household-sphere PEB (Path Coefficient = −0.047, *p* < 0.01), work-sphere PEB (Path Coefficient = −0.045, *p* < 0.01), public-sphere PEB (Path Coefficient = −0.059, *p* < 0.01). This suggests that higher EC reduces positive affective reactions, which in turn lessens pro-environmental behaviors. Finally, EC did not demonstrate a statistically significant indirect effect on Financial-sphere PEB via PEAR (Path Coefficient = −0.030, *p* = 0.052), indicating that perceived coping capacity may not meaningfully shape financial-sphere PEB through positive affective reactions.

**Table 6:** Indirect Effects Analysis

Indirect Paths	Effects	Standard Error	t-value	*p*-values	95% CI
ER → PEAR → HS-PEB	0.064*	0.028	2.280	0.023	0.019–0.127
ER →PEAR → WS-PEB	0.062*	0.025	2.461	0.014	0.018–0.117
ER → PEAR → PS-PEB	0.080**	0.029	2.763	0.006	0.030–0.143
ER → PEAR → FS-PEB	0.041	0.024	1.722	0.085	0.000–0.094
ER → NEAR → HS-PEB	0.078**	0.027	2.884	0.004	0.032–0.139
ER → NEAR → WS-PEB	0.082***	0.022	3.755	0.000	0.044–0.130
ER → NEAR → PS-PEB	0.087***	0.024	3.603	0.000	0.046–0.140
ER → NEAR → FS-PEB	0.045**	0.018	2.446	0.014	0.015–0.087
EC → PEAR → HS-PEB	−0.047**	0.016	2.918	0.004	−0.080 to −0.018
EC → PEAR → WS-PEB	−0.045**	0.016	2.777	0.005	−0.080 to −0.016
EC → PEAR → PS-PEB	−0.059**	0.019	3.081	0.002	−0.100 to −0.025
EC → PEAR → FS-PEB	−0.030	0.016	1.941	0.052	−0.062 to −0.000
EC → NEAR → HS-PEB	0.076**	0.026	2.911	0.004	0.032–0.135
EC → NEAR → WS-PEB	0.080**	0.023	3.450	0.001	0.030–0.143
EC → NEAR → PS-PEB	0.085**	0.026	3.304	0.001	0.041–0.142
EC → NEAR → FS-PEB	0.044**	0.016	2.744	0.006	0.017–0.080

ER = emergency relevance; EC = emergency coping; PEAR = positive environmental affective reactions; NEAR = negative environmental affective reactions; HS-PEB = household-sphere PEB; WS-PEB = work-sphere PEB; PS-PEB = public-sphere PEB; FS-PEB= financial-sphere PEB; SE= standardization error. * *p <* 0.05, **p < 0.01, ***p < 0.001.

**Fig. 2:**
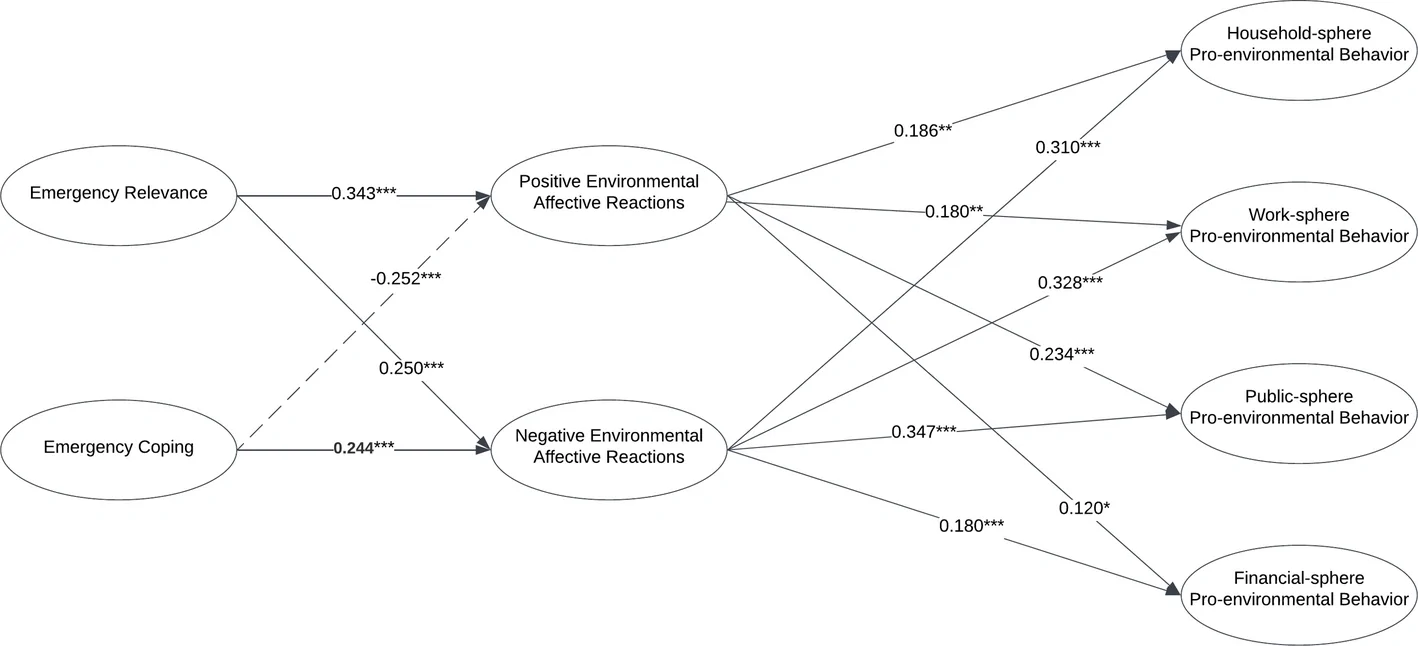
Structural Equation Model with Standardized Estimate Values. Dotted lines indicate unsupported hypotheses

In accordance with the results, the final structural equation model is presented in Fig. 2.

## Discussion

This study explored the impact of post-Covid-19 recession cognition on environmental affective reactions, and Pro-environmental Behavior (PEB) among Gen-Z in Pakistan. Our model shows that perceived relevance of an emergency and ability to cope with an emergency, acted through positive and negative affective reactions to influence PEBs across four different spheres.

The positive relationship between ER and both affective reactions highlights that increased emergency awareness can enhance individual’s motivation for environmental actions, aligning with the Affect Infusion Model [124], which suggests that the relevance of an emergency can induce negative affective responses in individuals, prompting them to adopt behaviors that alleviate the perceived threat. Conversely positive association of ER and NEAR in our study validate that negative emotional reactions, induced by environmental threats can lead to increased concern about environmental degradation, even though, if not addressed effectively, may also paralyze action, a reasoning also explicated by Leiserowitz [125].

We found that emergency coping negatively affected positive environmental affective reactions, indicating that as individuals feel more capable of coping with recession, their positive affective responses towards environment declines. This is consistent with previous studies for instance Waheed et al. [13], which suggests a similar pattern where in times of stress, individuals prioritize the conservation of their resources but when they have enough resources to cope with recession, they may experience reduced emotional urgency to engage in environmental causes, thus diminishing their positive affective reactions. It is important to note the contradiction of our findings with the study of Mi et al. [70], which revealed a positive influence of EC on PEAR during the COVID-19 pandemic. This discrepancy could be attributed to contextual differences; as during health emergencies like COVID-19, immediate personal health impact often leads to intense communal solidarity and PEAR as also noted by Xie et al. [126], while economic recessions mainly trigger financial stability concerns, causing individuals to prioritize economic survival over environmental considerations [13]. Moreover, generational difference of population can also explain this difference as Twenge et al. [127] suggest that economic uncertainty disproportionately impacts younger generations. This contradiction is significant as it underscores the varying psychological responses elicited by different emergency and generation types, stressing the need for considering demographic factors and tailored approaches in promoting environmental behaviors during health versus economic emergencies. This distinction is also critical because it extends prior crisis-based environmental behavior research [70] by demonstrating that different types of emergencies activate distinct affective pathways, suggesting that the psychological mechanisms driving pro-environmental behavior may vary substantially across crisis contexts.

Simultaneously, emergency coping increased negative environmental affective reactions, a finding that warrants its own theoretical unpacking. While it may seem paradoxical that greater coping capacity elevates negative affect, this pattern becomes coherent when viewed through the lens of cognitive dissonance theory [128]. Individuals who perceive themselves as capable of managing economic recession may remain simultaneously aware of environmental degradation while prioritizing financial survival over environmental engagement. This value conflict, as Thøgersen [129] demonstrates, can threaten one’s moral self-concept and manifest as guilt, anxiety, and frustration directed toward environmental issues. This is further reinforced by Hobfoll’s Conservation of Resources theory [130], which holds that resource loss is disproportionately more salient than resource gain and that negative events elicit stronger affective, cognitive, and behavioral responses. Consequently, even when individuals successfully cope with recession, the perceived trade-off of neglecting environmental responsibilities may register as an emotionally costly resource loss. Moreover, individuals often suppress or avoid climate-related emotions such as anxiety and guilt as a coping mechanism, a strategy that, rather than neutralizing these emotions, can paradoxically intensify their underlying affective charge. So in aggregate, these mechanisms suggest that economic coping efficacy does not neutralize environmental affect; but heightens the emotional tension between self-preservation and environmental responsibility, producing elevated negative affective reactions as a psychological by-product of that unresolved conflict. At the same time, it is worth noting that while this guilt-laden and conflict-driven form of negative affect does contribute to elevated NEAR, it operates through a more psychologically burdened and ambivalent channel than the threat-driven negative affect arising from emergency relevance. At the same time, because this relationship emerged in a direction contrary to the original expectation, the mechanisms proposed here should be understood as a theoretically informed interpretation instead of a definitive explanation. Nevertheless, the finding points toward the possibility that coping efficacy during prolonged economic crises may not simply alleviate environmental emotions but may instead reconfigure them through tensions between economic self-preservation and environmental responsibility, a possibility that warrants further investigation across different crisis contexts.

The study also found that the NEAR were stronger than the PEAR. This result is similar to that reported in previous literature by Volkos and Symvoulakis [131] and Greenglass et al. [132], indicating that economic emergencies impact individual’s emotional landscape by significantly elevating their negative affective states due to heightened concerns about financial stability and resource scarcity. Taking this trend forward, the study also found that impacts of NEAR on predicting PEBs are collectively stronger than those of PEAR. This can be understood through the lens of Cialdini et al.’s Negative State Relief Model [133], which posits that individuals witnessing negative emotions are encouraged to engage in behaviors that attenuate their distress, including pro-environmental behavior. Moreover, Gen-Z, being more aware of climate change impacts than older generations, experiences higher levels of eco-anxiety as noted by Tsevreni et al. [134] and Hurri [135]. This eco-anxiety, compounded by economic strain, leads to concerns that if climate change exacerbates, it will further hinder their ability to tackle environmental issues in the future as highlighted by Waheed et al. [12]. That being said, along with NEAR, PEAR also surfaced significantly during the recession. This duality may be explained by the fact that during economic downturn, individuals may still find reasons to engage in PEBs. This is often impelled by a desire to contribute to solutions in addition to maintaining a sense of agency and optimism [136].

This study also discovered that compared to others, public-sphere PEBs were most strongly impacted by both PEAR and NEAR. This finding contradicts the trend in previous literature, where public-sphere PEBs did not emerge as the most presiding form of environmental action. For instance, during COVID-19 pandemic, Bouman et al. [137] and Mi et al. [70] found private sphere behaviors, like those in the household, often take precedence, potentially because the pandemic confined people to their homes, prompting a focus on immediate environmental and household practices. Similarly, in the context of an ecological crises such as California Drought, individuals adopted household water conservation measures, exhibiting less engagement in public-sphere pro-environmental behaviors [138]. This was because direct impacts of drought on immediate surroundings, made individual’s household actions more urgent than broader public advocacy. In our study, the economic recession may have enhanced Gen Z’s understanding of systemic challenges, making them prioritize public actions. Tajfel and Tuner’s Social Identity Theory [139] provides a theoretical framework for understanding this shift, pointing that Gen Z’s strong identification with socio-environmental movements [140] drives their engagement in public-sphere PEBs. This collective identity was likely reinforced by the recession, making public-sphere actions more appealing as a measure to tackle large-scale issues that resonate with Gen-Z’s values. These findings suggest that, unlike some previous crisis contexts [70, 137], economic recessions may be associated with greater public-facing environmental engagement among younger generations.

After public-sphere PEBs, affective reactions predicted strongest impact for work-sphere PEBs. An interpretation of this finding can be traced back to Pakistan’s technological infrastructure where digital transition in workspaces and educational institutes is still in its early stages [141]. A generational lag prevails, with senior authoritative generations adapted to a non-digital work landscape, establishing a disconnect with Gen-Z. Schroth [29] highlights that Gen-Z, who are digitally more literate than previous generations, often finds this mismatch frustrating, as they are more accustomed with innovative technology. One possible interpretation is that such institutional mismatches may contribute to stronger negative affective responses within workplace and educational settings, thereby amplifying the influence of NEAR relative to PEAR.

Finally, the weakest impacts of both environmental affective reactions were observed on financial-sphere PEBs. As per Lusardi et al. [142], financial stress likely dominates cognitive resources during economic recessions, diminishing the capacity to engage in non-essential behaviors, including pro-environmental behaviors. For Gen-Z, already marked by significant financial-anxiety [143], the pressures of a recession further exacerbate concentration on instant economic survival. This stress culminates in cognitive narrowing, where financial concerns overshadow environmental prioritization within the financial-sphere. Mullainathan and Shafir [144] suggested that during financial scarcity, individuals prefer immediate financial gains over long-term environmental sustainability. Consequently, even though Gen-Z may hold profound sustainability values, the immediacy of financial stress during recession minimizes the plausibility that these values will translate into financial-sphere PEBs. The significance of this finding lies in its novelty as affective reaction’s impact on financial-sphere PEBs has not been explored before in either normal conditions or during recessions. Thus, this finding contributes to the literature by revealing a previously underexplored dimension of environmental behavior, demonstrating that financial-sphere engagement remains particularly vulnerable to economic stress, even among environmentally conscious youth.

Synthesized together, these findings extend current understandings of crisis-driven environmental behavior by demonstrating how economic recession contexts reshape the affective mechanisms and behavioral domains through which Gen-Z engages with environmental action. The theoretical contributions emerging from these findings are discussed in detail in Sect.  7.1.

## Limitations and recommendations for future research

Despite its insights, the generalizability of the study may be limited by some aspects. Firstly, the cross-sectional design of the research limit the ability to infer causality. As economic recessions are dynamic events, exhibiting evolving impacts over time, a cross-sectional snapshot may not fully capture the changes in emotional and behavioral responses that could prevail at different recession phases. Future research should utilize longitudinal designs to explore how pro-environmental behaviors fluctuate as recession advances and how post-disaster effects further transform these behaviors.

Secondly, the use of purposive sampling, while suitable for targeting a specific demographic such as Gen-Z [145], may also instigate sampling bias and limit broader statistical generalizability. Although recruitment was diversified beyond environmental organizations alone, voluntary participation and respondent-driven snowballing may still have increased the likelihood of participation among individuals comparatively more receptive to environmental discussions. Thus, the findings should be interpreted with caution when making broader claims regarding Gen-Z populations as a whole. Future use of random or stratified sampling could allow greater generalizability of the findings across diverse populations.

While our current approach is bespeaking of Gen-Z, an increasingly large segment of the population, our findings may not generalize to other cohorts, who could have different reactions to economic pressure; therefore, comparative generational analyses can endow better understanding of how environmental behaviors differ across age-cohorts. Moreover, the current study did not exclude impacts of personal psychological characteristics to avoid overextending the survey and risking respondent fatigue, which could compromise data quality. Traits such as psychological resilience and neuroticism can dampen or amplify affective reactions [146]. Therefore, future research should integrate these personality factors to determine their interaction with emergency cognition to influence pro-environmental behaviors.

Finally, while the study centered around the post-COVID-19 economic recession, it is important to note that in Pakistan, this period also coincided with intense heat waves [147], an environmental stressor that could have independently amplified environmental affective reactions. Given the continuing global climate and biodiversity crises, this limitation is not unique to the Pakistan context. The overlapping nature of ecological and economic crises depict a complex context where it becomes difficult to isolate these simultaneous effects. Future research to disentangle which form of crisis exerts a stronger impact on ER and EC, and how these link to shape pro-environmental behaviors would further elucidate the dynamic relationship between emergencies and PEBs.

## Conclusion and implications

With this study we aimed to understand how the cognition of post-COVID-19 economic recession influences environmental affective reactions and, subsequently, pro-environmental behaviors among Gen-Z. We found that, in the context of recession, emergency relevance significantly enhances both positive and negative environmental affective reactions. The stronger effect on negative affective reactions underscores the potent role of negative affective reactions in driving environmental action during economic recessions. We found that emergency coping reduced positive affective reactions while intensifying negative affective reactions, suggesting that individuals who feel capable of managing economic pressures may still experience heightened negative affective reactions regarding the environment. Moreover, we found affective reactions to predict pro-environmental behaviors differently across spheres. Public-sphere behaviors were most strongly influenced, particularly by negative affective reactions, demonstrating Gen-Z’s community and activism-focused approach to environmental action. Further, we found evidence that positive and negative affective reactions influence household and work-sphere PEBs differently. Finally, the smallest effects were detected for financial-sphere PEBs, suggesting that aligning economic priorities with environmental concerns may be more arduous during recessions; emphasizing the difficulty in harmonizing financial stability with environmental actions in times of economic stress.

### Theoretical contributions and implications

From a theoretical perspective, this research extends Affective Events Theory beyond its conventional applications in organizational and health contexts into the economic-environmental nexus, demonstrating that macroeconomic shocks function as affective events with downstream consequences for sustainability engagement. The findings also clarify the dual-valence role of affect during crisis, demonstrating that negative emotions are not merely obstacles but can serve as powerful catalysts for collective environmental action, challenging the dominance of positive affect in prior behavioral theories. Furthermore, by modelling behaviors across multiple domains, the study contributes to theorizing pro-environmental behavior as differentiated rather than unitary, with affective pathways contingent upon the distinct social and material affordances of each sphere. A particularly novel contribution lies in the introduction and empirical substantiation of the financial-sphere of PEB for Gen-Z. While existing research and policy interventions tend to privilege engagement in other spheres, the financial dimension in youth has remained underexplored, often reflecting an implicit assumption that younger cohorts lack the influence or capacity to act as financial agents [43, 44]. By demonstrating how economic stress reshapes cost-benefit thresholds and narrows the channel through which affect translates into sustainable financial practices, this study challenges such assumptions and underscores the need to recognize youth financial perspectives within socio-economic and environmental planning. Finally, the findings contribute by speaking about the formation of youth environmental subjectivities under crisis by illustrating how recessions act as critical junctures in which affective salience reorganizes priorities and opens (or forecloses) trajectories of environmental commitment among Gen-Z.

### Practical implications

From a practical perspective, the results highlight the opportunities for crisis-responsive policy and planning that leverage, rather than suppress, the affective intensity of economic downturns. Campaigns and interventions should acknowledge anxiety, frustration, and uncertainty but channel these emotions into structured avenues of agency, such as youth climate councils, community forums, and participatory platforms that institutionalize young people as planners and decision-makers rather than passive targets. The differentiated sphere effects identified in this study suggest that strategies must be tailored: in the public sphere, resources should support civic platforms and grassroots initiatives; in households and workplaces, behavioral nudges and institutional incentives can embed sustainable practices; and in the financial sphere, where findings show weakest affective influence, planners must address structural barriers by reducing cost burdens through subsidies, green microfinance, renewable energy tariffs tailored to students and youth-oriented financial literacy schemes.

In aggregate, although situated in Pakistan, the study speaks more broadly to developing economies where economic fragility and climate vulnerability intersect. The insights position economic recessions not only as periods of constraint but as climacteric junctures for incorporating sustainability into planning frameworks in ways that make economic stabilization and environmental responsibility reinforce each other. Embedding the perspectives of Gen-Z, a cohort shown here to be especially affectively responsive to crisis, ensures that recovery planning is responsive to their priorities and capable of sustaining their environmental engagement both during and beyond episodes of crisis.

## Supplementary Information

Below is the link to the electronic supplementary material.

Supplementary Material 1

## Data Availability

Data available at doi:10.17632/vmft4d24h6.1 (https:/doi.org/10.17632/vmft4d24h6.1%20).
