# Supplementary material for: Post COVID 19 economic recession shaping affective pathways of Gen Z’s environmental behavior across spheres of action
Source: Discov Sustain. 2026 Jul 7;7(1):1583. doi: 10.1007/s43621-026-03982-4 (PMC13597632; doi:10.1007/s43621-026-03982-4)
Supplement: Supplementary file 1 [file 43621_2026_3982_MOESM1_ESM.docx]

**Supplementary Material**

**Post COVID 19 Economic Recession Shaping Affective Pathways of Gen Z’s Environmental Behavior across Spheres of Action**

**Appendix A: Complete Questionnaire with Constructs and Items**

**Table A1: Complete Questionnaire with Constructs and Items.** This table provides the full set of 32 items used in the study's questionnaire, grouped under their respective eight constructs. Measures were rated on a 5-point Likert scale ranging from 1 (strongly disagree) to 5 (strongly agree) except for variables measuring self-reported pro-environmental behaviors in all four spheres which ranged from 1(rarely) to 5 (always).

| **Constructs** | **Items** | **Content** |
| --- | --- | --- |
|  | ER_1 | Post COVID-19 Economic Recession has put a strain on my financial resources. |
|  | ER_2 | Post COVID-19 Economic Recession has made it difficult for me to succeed in my job or studies. |
| Emergency Relevance (ER) | ER_3 | Post COVID-19 Economic Recession hindered my achievement of important goals in my life. |
|  | ER_4 | Post COVID-19 Economic Recession is negatively affecting the country's environmental issues |
|  | EC_1 | I can cope well with the financial challenges brought by the current economic situation. |
|  | EC_2 | I can cope with the inconvenience in my work or study caused by the tough current economic situation. |
| Emergency Coping (EC) | EC_3 | I can overcome the inconvenience or difficulties in my life brought by the current economic situation. |
|  | EC_4 | I can still support environmental issues despite the tough current economic situation. |
|  | PEAR_1 | I am encouraged by the increased public attention to environmental sustainability despite Economic challenges. |
| Positive Environmental  Affective Reactions (PEAR) | PEAR_2 | I feel optimistic that the Economic difficulties following Covid-19 are encouraging more people to adopt environmentally friendly behaviors. |
|  | PEAR_3 | The current economic situation makes me proud of my past actions to protect the environment. |
|  | PEAR_4 | I am inspired by the resilience of nature and our ability to make positive environmental changes during these tough Economic times. |
|  | NEAR_1 | The current economic situation makes me fearful about the worsening environmental issues due to reduced attention and funding. |
| Negative Environmental  Affective Reactions (NEAR) | NEAR_2 | I feel anxious about the long-term environmental damage that might occur as a result of economic priorities overshadowing ecological concerns. |
|  | NEAR_3 | The Economic hardships make me feel guilty for not being able to prioritize environmental actions as much as I would like. |
|  | NEAR_4 | The Economic situation makes me frustrated with others who disregard environmental conservation in favor of Economic gain. |
|  | HS-PEB 1 | I use reusable bags instead of plastic ones when shopping |
| Household Sphere Pro- Environmental Behaviors (HS- PEB) | HS-PEB 2 | I recycle household waste. |
|  | HS-PEB 3 | I reduce water usage in my household. |
|  | HS-PEB 4 | I pay attention to conserving energy. |
|  | WS-PEB 1 | I perform my job duties or academic tasks in environmentally-friendly ways. |
|  | WS-PEB 2 | I participate in environmental protection activities or plans organized by my company or educational institution. |
| Work Sphere Pro- Environmental Behaviors (WS-  PEB) | WS-PEB 3 | I recommend to my colleagues or peers, more environmentally friendly way of workings. |
|  | WS-PEB 4 | I use sustainable modes of transportation to commute to work or my educational institution. |
| Public Sphere Pro-Environmental Behaviors (PS- PEB) | PS-PEB 1 | I express my views on an environmental issue to stakeholders or government officials. |
|  | PS-PEB 2 | I engage in public awareness campaigns about environmental issues |
|  | PS-PEB 3 | I share information about environmental issues on social media. |
|  | PS-PEB 4 | I participate in protests or movements advocating for environmental policies. |
|  | FS-PEB 1 | I make conscious decisions to invest in environmentally sustainable financial products or services. |
| Financial Sphere Pro-Environmental Behaviors (FS- PEB) | FS-PEB 2 | I prioritize spending on environmentally friendly products or services over alternatives that may have a higher environmental impact. |
|  | FS-PEB 3 | I advocate for policies or initiatives that promote financial incentives for pro-environmental behaviors at local or national levels. |
|  | FS-PEB 4 | I support businesses or organizations that demonstrate a commitment to environmental sustainability through their financial practices or investments. |
